# Comprehensive growth performance, immune function, plasma biochemistry, gene expressions and cell death morphology responses to a daily corticosterone injection course in broiler chickens

**DOI:** 10.1371/journal.pone.0172684

**Published:** 2017-02-24

**Authors:** Gamal M. K. Mehaisen, Mariam G. Eshak, Ahmed M. Elkaiaty, Abdel-Rahman M. M. Atta, Magdi M. Mashaly, Ahmed O. Abass

**Affiliations:** 1 Department of Animal Production, Faculty of Agriculture, Cairo University, Giza, Egypt; 2 Department of Cell Biology, National Research Centre, Giza, Egypt; Radboud Universiteit, NETHERLANDS

## Abstract

The massive meat production of broiler chickens make them continuously exposed to potential stressors that stimulate releasing of stress-related hormones like corticosterone (CORT) which is responsible for specific pathways in biological mechanisms and physiological activities. Therefore, this research was conducted to evaluate a wide range of responses related to broiler performance, immune function, plasma biochemistry, related gene expressions and cell death morphology during and after a 7-day course of CORT injection. A total number of 200 one-day-old commercial Cobb broiler chicks were used in this study. From 21 to 28 d of age, broilers were randomly assigned to one of 2 groups with 5 replicates of 20 birds each; the first group received a daily intramuscular injection of 5 mg/kg BW corticosterone dissolved in 0.5 ml ethanol:saline solution (CORT group), while the second group received a daily intramuscular injection of 0.5 ml ethanol:saline only (CONT group). Growth performance, including body weight (BW), daily weight gain (DG), feed intake (FI) and feed conversion ratio (FC), were calculated at 0, 3 and 7 d after the start of the CORT injections. At the same times, blood samples were collected in each group for hematological (TWBC’s and H/L ratio), T- and B-lymphocytes proliferation and plasma biochemical assays (total protein, TP; free triiodothyronine hormone, fT_3_; aspartate amino transaminase, AST; and alanine amino transaminase, ALT). The liver, thymus, bursa of Fabricius and spleen were dissected and weighed, and the mRNA expression of insulin-like growth factor 1 gene (IGF-1) in liver and cell-death-program gene (caspase-9) in bursa were analyzed for each group and time; while the apoptotic/necrotic cells were morphologically detected in the spleen. From 28 to 35 d of age, broilers were kept for recovery period without CORT injection and the same sampling and parameters were repeated at the end (at 14 d after initiation of the CORT injection). In general, all parameters of broiler performance were negatively affected by the CORT injection. In addition, CORT treatment decreased the plasma concentration of fT_3_ and the mRNA expression of hepatic IGF-1. A significant increase in liver weight accompanied by an increase in plasma TP, AST and ALT was observed with CORT treatment, indicating an incidence of liver malfunction by CORT. Moreover, the relative weights of thymus, bursa and spleen decreased by the CORT treatment with low counts of TWBC’s and low stimulation of T & B cells while the H/L ratio increased; indicating immunosuppressive effect for CORT treatment. Furthermore, high expression of caspase-9 gene occurred in the bursa of CORT-treated chickens, however, it was associated with a high necrotic vs. low apoptotic cell death pathway in the spleen. Seven days after termination of the CORT treatment in broilers, most of these aspects remained negatively affected by CORT and did not recover to its normal status. The current study provides a comprehensive view of different physiological modulations in broiler chickens by CORT treatment and may set the potential means to mount a successful defense against stress in broilers and other animals as well.

## Introduction

Broiler production forms an essential component to meet the increasing demand of animal protein in either the developed or developing countries. The modern large-scale broiler production provokes a new stressful situation around broilers due to thermal conditions, high-stocking density, immunological challenge, handling, transportation and feed quality. All kinds of such stressors can elicit the biological response of broiler chickens to re-establish homeostatic conditions [1], and directly or indirectly activate the hypothalamic-pituitary-adrenal (HPA) axis [2]. The activated HPA axis results in the release of adrenocorticotropic hormone (ACTH), which stimulates the adrenals to secret and release glucocorticoids, including corticosterone (CORT), into blood circulation [1]. In chickens, the high levels of CORT in blood circulation is associated with a marked regression in the growth performance, feed efficiency and fuel metabolism [3–8]. The weight and function of the lymphoid organs including thymus, bursa of Fabricius and spleen also decreased by the CORT treatment in birds [3,4,9]. In contrast, the effect of CORT on the liver is anabolic and the fat pads are grossly enlarged in liver of broilers treated with CORT [6].

It was found that exposure to stressors elevates the blood concentration of heterophils (the avian counterpart to the mammalian neutrophils) and depresses the blood concentration of lymphocytes [10]. As an important immune parameter, the ratio of circulating heterophils to lymphocytes (H/L ratio) is one of the most recognizable symptoms of stress in chickens [11–14]. The administration of exogenous corticosterone resulted in an increase in the plasma corticosterone concentration and H/L ratio with a global decrease in the circulating leukocyte number in young chickens [9,13,14]. On the other hand, lymphocytes in birds are specifically separated into T and B cells during the development of the thymus and bursa of Fabricius glands, respectively. Such cells carry receptors for stress hormones produced by the adrenal and pituitary glands, so it could be used as another index to stress [9]. Particularly, high concentrations of glucocorticoids have immunosuppressive effects by inhibiting, for example, antibody production from B cells, T cell proliferation and phagocytosis [15,16]. In addition, some blood physiological and biochemical changes may occur in the stress- and/or CORT-exposed broiler chickens and may be responsible for their low growth rates [17]. In a recent study, the CORT treatment enhanced the total plasma protein levels in broiler chickens [3]. It was previously found that corticosterone increased protein degradation as indicated by the reduction in skeletal RNA/protein synthesis [18] and the rising in the muscle proteolysis [19]. The increase in aspartate and alanine amino transaminase (AST and ALT) enzymes levels usually appear in the blood when there is tissue impairment or liver malfunctioning caused by excessive stress [20]. Two other components are mediated by each other and strongly correlated with the growth performance in young chickens; triiodothyronine hormone (T_3_) and insulin-like growth factor 1 (IGF-1) [21,22]. It has been reported that the decreased performance in stressed broilers is mainly caused by the reduction in T_3_ and thyroxin (T_4_) levels [23]. Many studies have also reported the negative effects of environmental and nutritional stress on the expression of IGF-1 gene and its circulating levels in different avian species during embryonic and post-hatch growth and development [24–26]. It was clearly reported that glucocorticoid administration in chickens has a suppressive effect on the plasma T_3_ levels [27,28] and the circulating concentrations of hepatic IGF-1 [29].

Rearing stress conditions and physical agents can also activate apoptosis; a mode of cell death that occurs under normal physiological conditions and is triggered by one or more of intracellular factors [30–32]. The apoptotic morphology, including plasma membrane blebbing, cytoplasm vacuolization, chromatin condensation and DNA degradation, is essentially the result of the proteolytic action of caspases upon specific cellular substrates [33,34]. Caspase cascades are responsible for both initiating and amplifying early apoptotic signals (e.g. caspase-1, -2, -8, -9, -10) as well as executing apoptosis (e.g. caspase-3, -6, -9) [35,36]. It has been well documented that the chronic stress may influence the expression of caspase genes in broiler chickens and other vertebrates [37]. In some cases, stressful stimuli can initiate the necrotic pathway form of cell death programs, a process that has distinct morphological features; it is accompanied by a rapid collapse of plasma membrane, and it is independent of expression of new genes [38]. In one of few studies that differentiated between apoptosis and necrosis pathways in rat’s Leydig cells [39] and postnatal neuron cells [40] cultured with CORT, a higher necrotic than apoptic cells were detected in groups with higher CORT concentration.

Most researchers have focused on the effect of stressors itself and/or stress-related glucocorticoids administration on specific pathways related to stress in birds. However, the administration of corticosterone to broiler chickens simply serves as a practical, controlled and flexible tool in the research of their adaptation to stress. In the present study, the research was to set forth an overall view of modulation induced by corticosterone exposure as simulation for stress condition in broiler chickens. Hence, this work aimed to assess comprehensive responses related to broiler performance, immune function, plasma biochemistry, gene expression and cell death morphology throughout a 7-day course of daily CORT injections. In addition, all measurements were repeated at 7 d after termination of the 7-day course of the CORT treatment to estimate the capability of broiler chickens for recovery to normal physiological status after overriding the stress.

## Materials and methods

### Birds, treatment and ethical statement

The current study was conducted in the Poultry Services Center at the Faculty of Agriculture, Cairo University. A total of 200 one-day-old commercial broiler chicks (Cobb500^™^) were obtained from a commercial hatchery and housed on a deep litter floor brooder. Ambient temperature on d 1 was set at 33°C and then was gradually reduced until 24°C by d 21. The light regimen was 23L:1D. Chicks were fed commercial broiler diets according to the recommendations of the National Research Council (NRC, 1994). Water and feed were supplied *ad libitum* during the study.

From 21 to 28 d of age, birds were randomly assigned to one of 2 groups with 5 replicates of 20 birds per replicate. Broilers of the first group received a daily intramuscular injection (at 10:00 AM) of corticosterone (cat# C2505, Sigma, St. Louis, MO 63103, USA) adjusted based on the average body weight of birds at dose of 5 mg/kg; dissolved in 0.5 ml ethanol:saline (1:1, vol/vol) solution (CORT group). The second group received a daily intramuscular injection of 0.5 ml ethanol:saline (1:1, vol/vol) solution and served as positive control (CONT group). Growth performance of broilers was recorded during the experimental period as detailed later. At 21 d of age, before CORT treatment (0 d of injection), blood samples were collected from 5 birds per treatment group (one per replicate) via the wing vein for determination of hematological assay and lymphocyte proliferation. Another 5 birds from each group were sampled and plasma was obtained for biochemical analyses. In addition, 5 birds from each treatment group were sacrificed, by cervical dislocation, and the liver, thymus, bursa of Fabricius and spleen were harvested, weighed and kept on ice until the following analyses. The mRNA expression of insulin-like growth factor 1 gene (IGF-1) was analyzed in liver tissues, while the expression of cell-death-program gene (caspase-9) was analyzed in bursa tissues. Finally, spleen tissues were subjected to a morphological detection of apoptotic/necrotic cells under fluorescent microscope. The same sampling procedures and parameters were repeated during the treatment at 3 and 7 d after the start of CORT injection (at 24 and 28 d of age). After that, birds were kept for recovery period without CORT injection and the same parameters were measured 7 d later (at 14 d after the start of CORT injection = 35 d of age). Each bird in the group was sampled only once and then was removed from the experiment.

Birds were monitored closely, twice a day, to detect any signs of stress (breathing difficulty, watery discharge of the peak, decreased appetite, ruffled feathers, or droopy looking) throughout the experimental period. Accordingly, when one or more of these signs appeared, cervical dislocation was used to end the life of these birds. This process was accomplished to minimize suffering of birds and to allow humane endpoints. All experimental protocols were approved by Cairo University Ethics Committee for the Care and Use of Experimental Animals in Education and Scientific Research (CU-IACUC).

### Growth performance and organs weight

Individual body weights (BW) were recorded at 0, 3 and 7 d after the start of CORT injection course (21, 24 and 28 d of age), and at 7 d after cessation of the 7-day course of the CORT treatment (14 d after initiation of CORT injection; 35 d of age) for each group. The average daily gain (DG), feed intake (FI) and feed conversion ratio (feed/gain; FC) for each replication in the group were obtained at periods 0–3 d and 3–7 d of the CORT treatment course and at 7–14 d of the recovery period (21–24, 24–28 and 28–35 d of age, respectively). After cervical dislocation of broilers, the weights of liver, thymus, bursa of Fabricius and spleen were also calculated relatively to the body weights in each group at 0, 3 and 7 d after initiation of the CORT injection, and after 1 week of recovery period.

### Hematological assay

Whole blood samples of approximately 3 ml were collected using heparinized syringes and transferred immediately to heparinized tubes. The total white blood cells (TWBC’s) were manually determined by mixing 490 μl of brilliant cresyl blue dye with 10 μl of whole blood sample, and then the total leukocytes were counted under a microscope at a magnification of 200x using a hemocytometer slide [41]. The H/L ratio was determined according to Zhang *et al*. [42] with modification. In brief, a droplet of blood (5 μl) was used to make smears on a clean glass slide (2 slides per each sample). The smears were stained with Hema-3 (cat# 22–122911, Fisher scientific, USA) after drying and fixing with methyl alcohol. On each slide, heterophils and lymphocytes were counted under a microscope at magnification of 1000x with oil immersion until a total of 100 cells were reached. After averaging the cells of 2 slides, the ratios of heterophils to lymphocytes were calculated.

### Lymphocyte proliferation

The T and B lymphocyte proliferation assays were done according to methods described by Zhang and Guo [43] with some modifications. The heparinized blood samples were added to separation medium Histopaque-1077 (cat# 10771, Sigma, USA), and were then centrifuged at 1030 xg for 20 min at 4°C. Peripheral blood mononuclear cells (PBMCs) were isolated and washed twice with RPMI-1640 (Invitrogen Corp., Grand Island, NY, USA) incomplete culture medium, and then re-suspended in 2 ml of RPMI-1640 complete culture medium. The viable lymphocytes were detected using Trypan Blue dye and plated in triplicate wells (96-well plate) at 1×10^6^ cells per well. Then, 50 μl of either Concanavalin-A (Con-A, 45 μg/ml, cat# C5275, Sigma, USA) or Lipopolysaccharide (LPS, 10 μg/ml, cat# L4391, Sigma, USA) was added to selected wells to induce the proliferation of T lymphocyte and B lymphocyte, respectively; while control wells received 50 μl of RPMI-1640 medium. Cells were then incubated for 48 h at 42°C with 5% CO_2_. After incubation, 15 μl of 3-[4,5-dimethylthiazol]-2,5-diphenyltetrazolium bromide (MTT, 5 mg/ml, cat# M2128, Sigma, USA) was added to each well and the cells were incubated for another 4 h. Subsequently, 100 μl of 10% sodium dodecyl sulfate dissolved in 0.04 M HCl solution was added to each well to lyse the cells and solubilize the MTT crystals. Finally, the absorbance at 570 nm was recorded using an automated ELISA microplate reader (ChroMate Microplate Reader-4300, Awareness Technology Inc., Palm City, FL, USA). Stimulating index (SI) for T and B cells was calculated as follows: SI = OD570_(stimulated cells)_ / OD570_(unstimulated cells)_.

### Plasma biochemical analyses

Blood samples of approximately 3 ml were withdrawn from the brachial wing vein in heparinized tubes and centrifuged at 1030 xg for 10 min at 4°C. The plasma was separated and stored at -20°C until analyzed. The plasma levels of total protein (TP), AST and ALT were analyzed by automatic scanning spectrophotometer (CE1010, Cecil Instruments Limited, Cambridge, United Kingdom) using colorimetric assay kits (TP-2020 for TP, and AT-1034(45) for both AST and ALT; Biodiagnostic Inc, Dokki-Giza, Egypt). The fT_3_ was measured by the automated ELISA microplate reader using enzyme immunoassay ELISA kits (EIA-10301, Chemux BioScience Inc, San Francisco, CA, USA). The standard curves and calculations, as well as accuracy and sensitivity of the assay were performed following the kits protocol for each assay.

### Quantitative real-time PCR analysis

Total RNA was extracted from liver and bursa tissues using the standard TRIzol Reagent extraction method. RNA was dissolved in diethylpyrocarbonate (DEPC)-treated water by passing solution a few times through a pipette tip. Total RNA was treated with 1 U of RQ1 RNase-free DNase (Invitrogen, Germany) to digest DNA residues, then re-suspended in DEPC-treated water. The purity of total RNA was assessed spectrophotometrically at 260/280 nm, and the integrity of extracted RNA was determined by using 1.5% agarose gel electrophoresis. Then total RNA was reverse-transcribed into cDNA by using RevertAidTM First Strand cDNA Synthesis Kit (MBI Fermentas, Germany) according to the manufacturer's directions. Briefly, an amount of total RNA (5μg) was used with a reaction mixture, termed as master mix (MM). The MM consisted of 50 mM MgCl_2_ and 5x reverse transcription (RT) buffer (50 mM KCl, 10 mM Tris-HCl, pH 8.3, 10 mM dNTPs, 50 μM oligo-dT primers, 20 U ribonuclease inhibitor and 50 U M-MuLV reverse transcriptase). The RT reaction was carried out at 25°C for 10 min, followed by 1 h at 42°C, and the reaction was stopped by heating for 5 min at 99°C. Afterwards, the reaction tubes containing cDNA were stored at -20°C until being used for quantitative real time-polymerase chain reaction (qRT-PCR).

PCR reactions were set up in 25 μl reaction mixtures containing 12.5 μl of 1× SYBR Premix Ex TaqTM (TaKaRa, Biotech. Co. Ltd., Germany), 0.5 μl sense primers (0.2 μM), 0.5 μl antisense primer (0.2 μM), 6.5 μl distilled water, and 5 μl of cDNA template. The reaction program was allocated to 3 steps of thermal cycling parameters. The first step was set to 95.0°C for 3 min. The second step consisted of 40 cycles in which each cycle was divided to 3 steps: (a) at 95°C for 15 sec, (b) at 55°C for 30 sec, and (c) at 72°C for 30 sec. The last step consisted of 71 cycles which started at 60°C and then increased by 0.5°C every 10 sec up to 95°C. At the end of each qRT-PCR, a melting curve analysis was performed at 95°C to check the quality of the used primers. Each experiment included a distilled water control.

The qRT-PCR of IGF-1and caspase-9 genes were normalized to the main expression of ß-actin and transformed using the comparative cycle threshold (CT) method to quantify expression levels as previously described by Ellestad *et al*. [44]. Sequence-specific primers (Table 1) for the real-time PCR were designed using the Primer blast web interface (http://www.ncbi.nlm.nih.gov/tools/primer-blast/index.cgi).

**Table 1:** Details of primers used for real-time PCR quantitative analysis.

Gene symbol	GenBank accession no.	Primer sequences (5'->3')	Product size (bp)	Melting temperature (°C)
**IGF-1**	FJ977570.1	F: CACCTAAATCTGCACGCT	140	55
		R: CTTGTGGATGGCATGATCT		
**Caspase-9**	XM_424580.2	F: TCCCGGGCTGTTTCAACTT	208	60
		R: CCTCATCTTGCAGCTTGTGC		
**ß-actin**	NM205518	F: TGCGTGACATCAAGGAGAAG	300	58
		R: TGCCAGGGTACATTGTGGTA		

### Morphological detection of cell death categories

Apoptotic changes in the spleen samples were determined morphologically by fluorescent microscope after labeling with acridine orange and ethidium bromide (AO/EB) dyes [45]. Briefly, spleen tissues were washed in PBS (Sigma), chopped finely and centrifuged at 9220 xg for 5 min. The pellet obtained was suspended in trypsin-EDTA solution (0.25%, 53 mM; cat#T4049, Sigma) in PBS for 1 hr at 37°C and smeared on clean glass slides. Finally, spleen cells smears were air-dried and fixed in a solution of methanol/acetic acid (3:1). The slides were stained with 25 μl of AO/EB dye mixture (4μg/ml AO (cat#A9231, Sigma) and 4μg/ml EB (cat#E7637, Sigma) in PBS, pH 7.4). The slides were examined under fluorescence microscope using B2A filter (450–490 nm excitation, 515 nm emission, at 100x magnification, Nikon Tokyo, Japan), and connected to a COHU 4910 video camera (Cohu, Inc., San Diego, CA, USA) and a personal computer—based image analysis system (Lucia-Comet v.4.51). The cells were divided into three categories as follows: viable cells (green colored), apoptotic cells (yellow colored), and necrotic cells (red colored). A total of 100 cells were counted, and the % of total apoptotic and necrotic cells were examined for each experimental group (CONT 0, 3, 7 and 14; CORT 0, 3, 7 and 14). Detailed information on the cell death categories and its rates in each experimental group has been demonstrated in (S1 Table). Also, visual examples for apoptotic categories in each experimental group are represented in (S1 Fig).

### Statistical analysis

All statistical analyses were performed using IBM SPSS 22.0 software package (IBMcorp., NY, USA, 2013). A general linear model (GLM) procedure was performed to analyze all data including CORT treatment (CORT and CONT groups), time (0, 3, 7 and 14 d of the course of the treatment) and their interactions as fixed effects. When interactions between main effects were significant, a multiple pairwise comparison among multiple means within CORT treatment group were made by Duncan’s test and the differences between CONT and CORT groups within each time was also performed using independent-samples T-test. Values were considered statistically different at P <0.05 and results were expressed as least square means ± standard error.

## Results

### Growth performance

**Fig 1:**
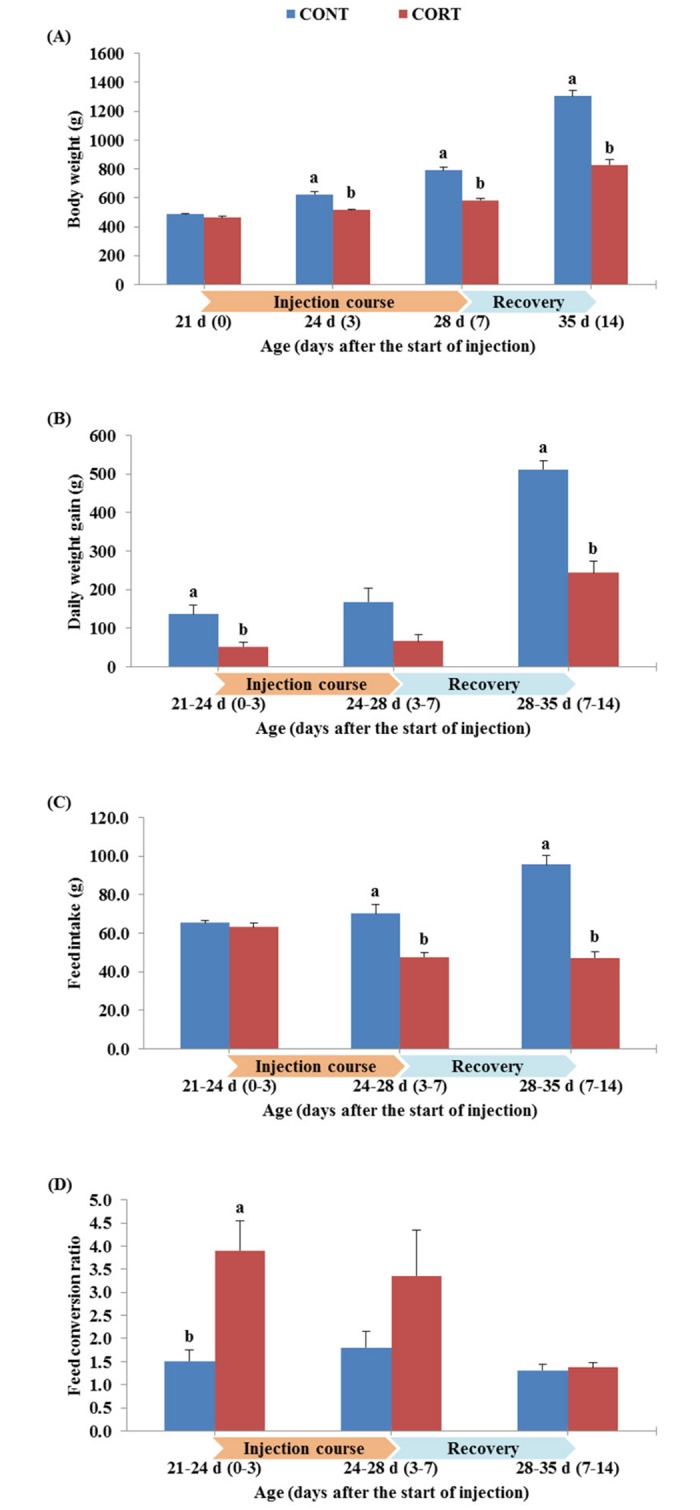
Effect of a 7 d course of daily saline (CONT) or corticosterone at dose of 5 mg/kg BW (CORT) injections on the body weight (A), daily gain (B), feed intake (C) and feed conversion (D) of broiler chickens during the treatment course (0, 3 and 7 d after the start of injection; 21, 24 and 28 d of age) and one week after cessation of the treatment (14 d after the start of injection; 35 d of age). Bars express the mean ± SEM (n = 36). Bars of CONT and CORT groups, within each time of the treatment course, with different letters (a, b) are significantly different at P<0.05.

The effect of the 7 d course of daily corticosterone injections on the BW, DG, FI and FC of broiler chickens during the treatment course and after the post-treatment recovery period is shown in Fig 1. The corticosterone injection, significantly, decreased the BW of broiler chickens at 3 and 7 d of the course of the treatment when compared with the control group (516 g vs. 624 g and 584 g vs. 792 g BW for CORT vs. CONT group at 3 and 7 d of the treatment course, respectively), and it was still low after recovery (828 g vs. 1303 g BW of CORT vs. CONT chickens at 14 d of the treatment course, Fig 1-A). A significantly (P<0.05) lower DG was observed for CORT-treated chickens compared to the CONT group during the first 3 days from the start of the CORT injection (52 g vs. 136 g DG during 21–24 d of age). This was also observed during the recovery period (244 g vs. 512 g DG during 28–35 d of age) (Fig 1-B). The FI estimated for CORT-treated chickens was significantly lower than the CONT during 3–7 d from the start of the corticosterone treatment (47.8 vs. 70.2 g FI/chicken/day during 24–28 d of age) and also during the recovery period (47.3 vs. 95.7 g FI/chicken/day during 28–35 d of age) (Fig 1-C). In contrast, the FC was significantly higher in chickens of CORT group than in those of CONT group only within 3 days from the start of the corticosterone injection (3.9 vs. 1.5 FC during 21–24 d of age, Fig 1-D).

### Organs weight

**Fig 2:**
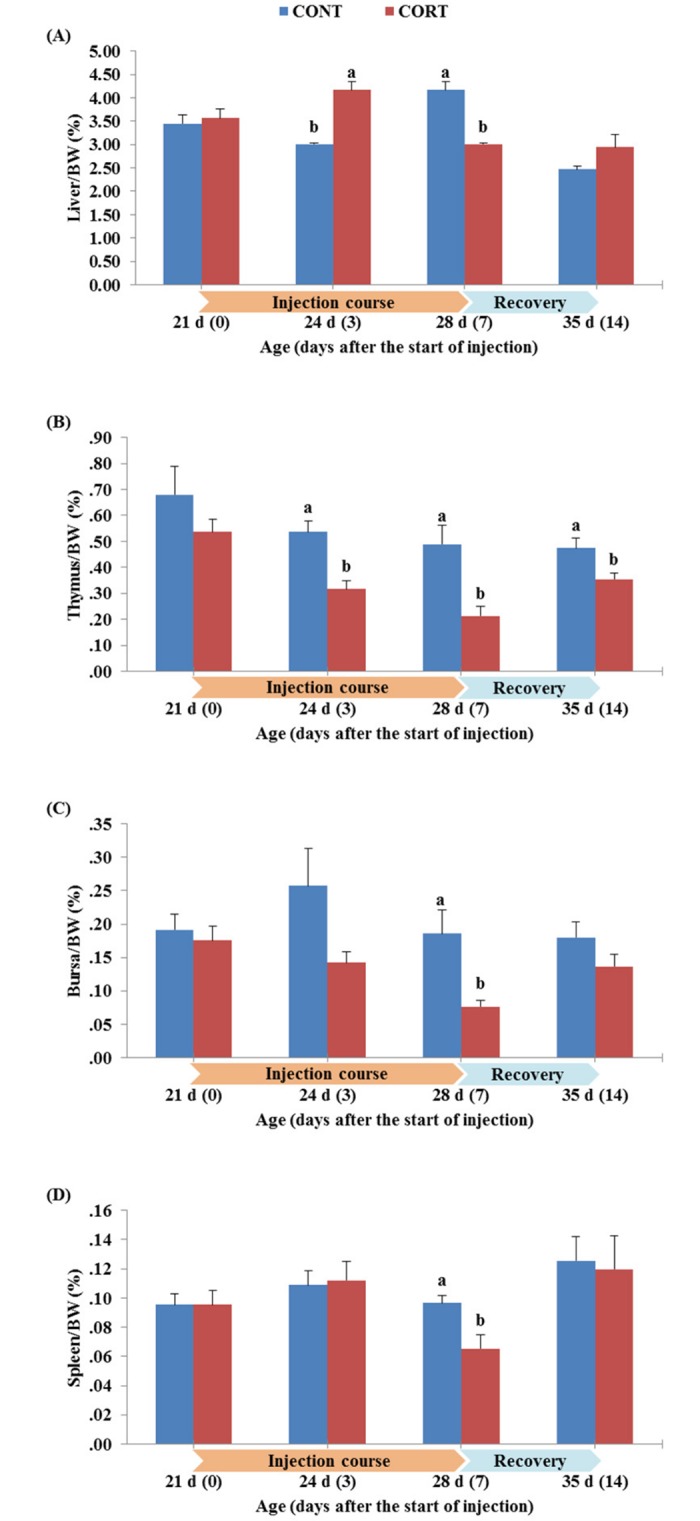
Effect of a 7 d course of daily saline (CONT) or corticosterone at dose of 5 mg/kg BW (CORT) injections on the relative weights of liver (A), thymus (B), bursa (C) and spleen (D) to the body weights of broiler chickens during the treatment course (0, 3 and 7 d after the start of injection; 21, 24 and 28 d of age) and one week after cessation of the treatment (14 d after the start of injection; 35 d of age). Bars express the mean ± SEM (n = 5). Bars of CONT and CORT groups, within each time of the treatment course, with different letters (a, b) are significantly different at P<0.05.

The effect of the 7 d course of daily corticosterone injection on the organs relative weights of broilers during the treatment course and after the post-treatment recovery period is shown in Fig 2. The relative liver weight of broilers in CORT group (4.17%) was significantly (P<0.05) higher than that in CONT group (3.01%) at both 3 and 7 d after the start of treatment (Fig 2-A). As shown in Fig 2-B, the relative weight of thymus glands in treated chickens was significantly (P<0.05) lower than that in the control chickens during the 7-d course of treatment (0.32% vs. 0.54% and 0.21% vs. 0.49% for CORT vs. CONT at 3 and 7 d after the start of injection, respectively). The same trend was also observed at the end of the recovery period (0.35% for CORT vs. 0.47% for CONT at 14 d after the start of injection). The relative weight of bursa was lower in CORT group than in CONT group (Fig 2-C); however, this decrease was significant (P<0.05) only at 7 d after the start of injection (0.08% vs. 0.18% for CORT vs. CONT group, respectively). A significantly lower relative spleen weight was observed in treated chickens (0.07%) compared to the controls (0.10%) at 7 d after the start of the CORT injection (Fig 2-D).

### Hematological assay

**Fig 3:**
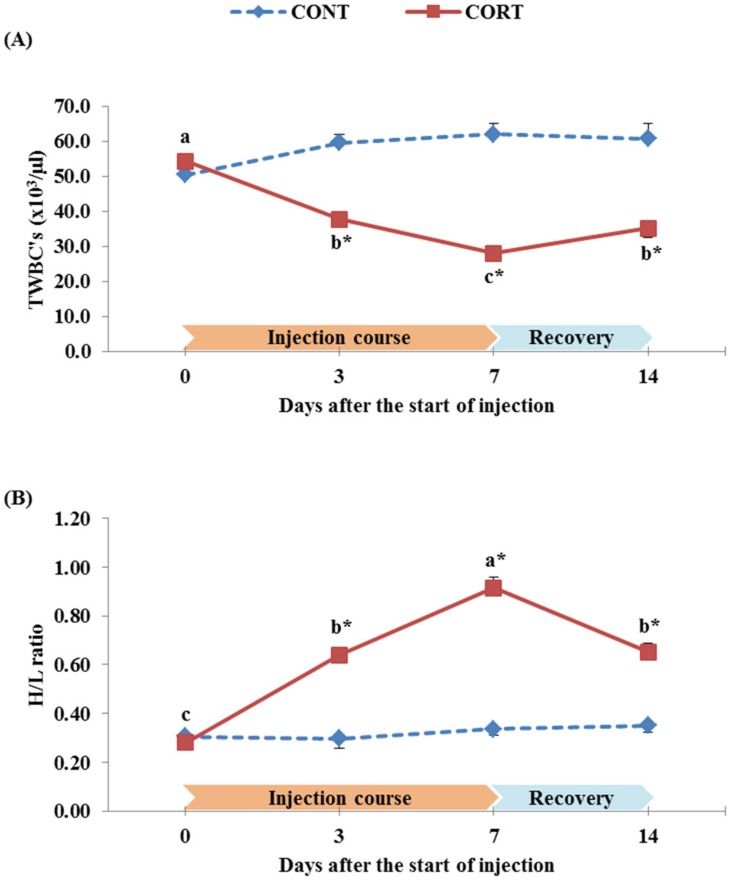
Effect of a 7 d course of daily saline (CONT) or corticosterone at dose of 5 mg/kg BW (CORT) injections on the TWBC’s (A) and the heterophil to lymphocyte (H/L) ratio (B) of broiler chickens during the treatment course (0, 3 and 7 d after the start of injection) and one week after cessation of the treatment (14 d after the start of injection). Data express the mean ± SEM (n = 5). Means within a treatment group with different letters (a, b, c) are significantly different at P<0.05.*Significant difference between treatment groups within each time of the treatment course (P<0.05).

The effect of the 7 d course of daily corticosterone injections on the TWBC’s and the H/L ratio of broiler chickens during the treatment course and after the post-treatment recovery period is presented in Fig 3. The corticosterone treatment significantly decreased the TWBC’s at 3 d (37.8 vs. 59.6 x10^3^/μl for CORT vs. CONT group) and 7 d (28.0 vs. 62.1 x10^3^/μl for CORT vs. CONT group) of the course of the treatment (Fig 3-A). Seven days following the termination of CORT injections, the TWBC’s increased but still lower than that observed at the start of the treatment (35.2 vs. 60.8 x10^3^/μl for CORT vs. CONT group). In contrast, the H/L ratio was significantly (P<0.05) higher due to corticosterone treatment course after 3 d (0.64 vs. 0.30 for CORT vs. CONT group) and 7 d (0.91 vs. 0.34 for CORT vs. CONT group) of the CORT injections (Fig 3-B), while it decreased after cessation of the CORT injections (0.65 for the CORT group vs. 0.35 for the CONT group at 14 d after the start of the treatment).

### Lymphocyte proliferation

**Fig 4:**
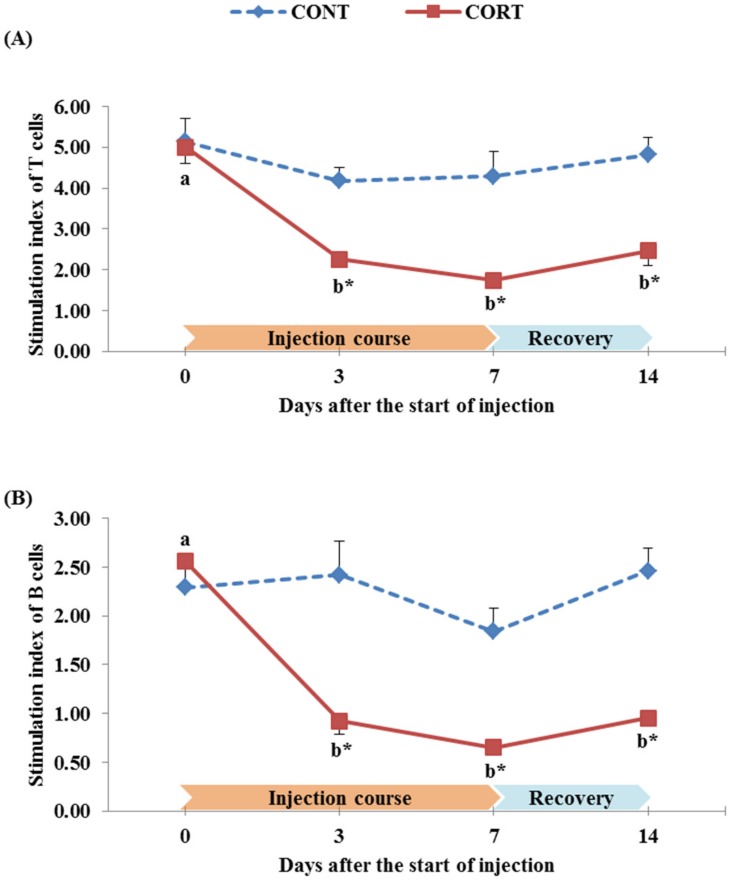
Effect of a 7 d course of daily saline (CONT) or corticosterone at dose of 5 mg/kg BW (CORT) injections on the stimulation index of T cells (A) and B cells (B) of broiler chickens during the treatment course (0, 3 and 7 d after the start of injection) and one week after cessation of the treatment (14 d after the start of injection). Data express the mean ± SEM (n = 5). Means within a treatment group with different letters (a, b, c) are significantly different at P<0.05.*Significant difference between treatment groups within each time of the treatment course (P<0.05).

The effect of a daily injection course of corticosterone over 7 days followed by a 7 days post-treatment recovery period on the lymphocyte proliferation of broiler chickens is illustrated in Fig 4. The stimulation index (SI) in broiler chickens of the CONT group ranged from 4.17 to 5.14 for T cells (Fig 4-A) and from 1.84 to 2.46 for B cells (Fig 4-B). The CORT treatment significantly (P<0.05) decreased the SI of T cells from 5.01 at the start of injection course to 2.25 and 1.74 at 3 and 7 d after the start of injection course, respectively (Fig 4-A). The SI of B cells was also decreased (P<0.05) by the CORT treatment from 2.56 at the start of injection course to 0.92 and 0.65 at 3 and 7 d after the start of injection course, respectively (Fig 4-B). One week after termination of the CORT injection, the lymphocyte proliferation remained significantly (P<0.05) lower in the treated chickens than the control chickens (SI of T cells: 2.47 vs. 4.82 (Fig 4-A); SI of B cells: 0.96 vs. 2.46 (Fig 4-B); for CORT group vs. CONT group at 14 d after the start of injection course).

### Plasma biochemical analyses

**Fig 5:**
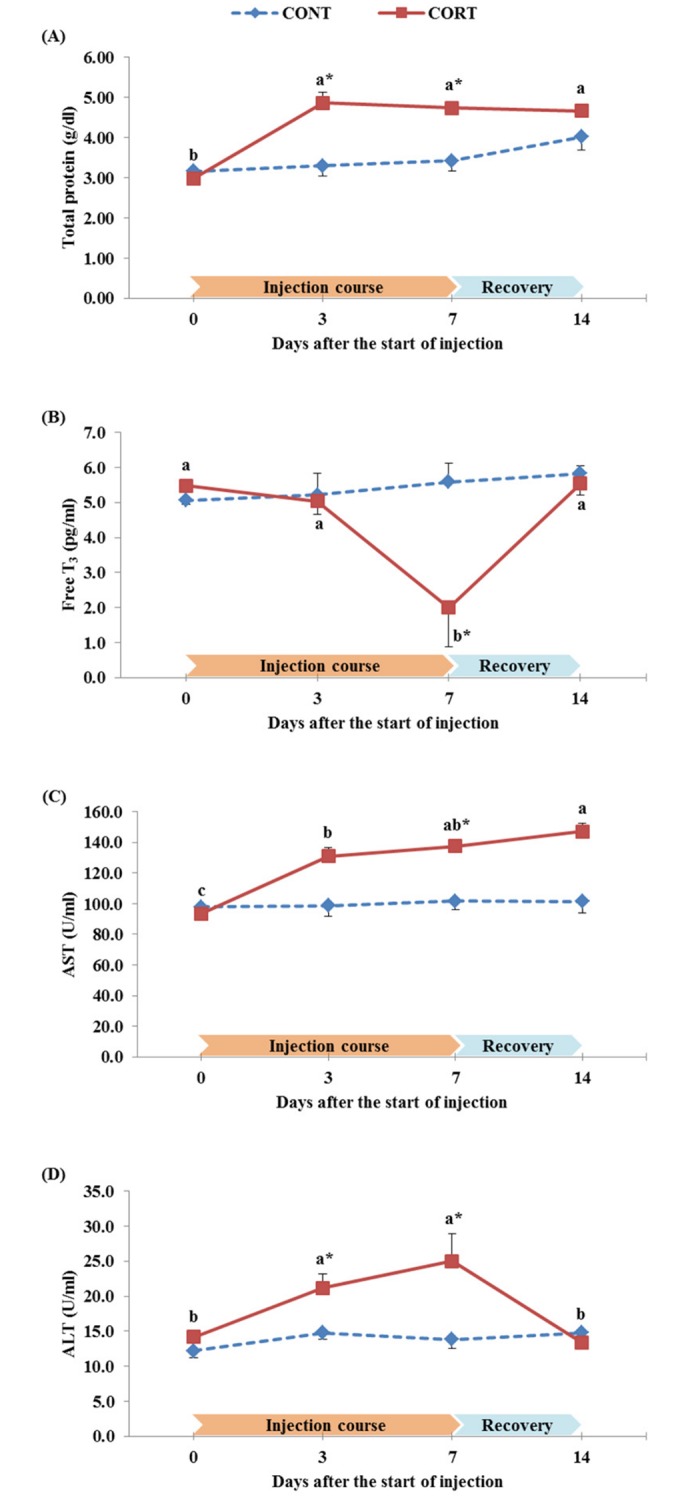
Effect of a 7 d course of daily saline (CONT) or corticosterone at dose of 5 mg/kg BW (CORT) injections on the plasma concentration of total protein (A), free T_3_ hormone (B), AST (C) and ALT (D) in broiler chickens during the treatment course (0, 3 and 7 d after the start of injection) and one week after cessation of the treatment (14 d after the start of injection). Data express the mean ± SEM (n = 5). Means within a treatment group with different letters (a, b, c) are significantly different at P<0.05.*Significant difference between treatment groups within each time of the treatment course (P<0.05).

The effect of a daily corticosterone injections at dose of 5 mg/kg BW over 7 days followed by one week of recovery period on the plasma concentration of TP, fT_3_, AST and ALT in broiler chickens is presented in Fig 5. In the CORT group, the TP was significantly higher during the treatment course (4.86 and 4.74 g/dl at 3 and 7 d after the start of injection, respectively) and after the recovery period (4.66 g/dl at 14 d after the start of injection) when compared to the 0 d of injection (2.98 g/dl; Fig 5-A). When compared to the CONT group, the TP was significantly (P<0.05) higher in CORT group at 3 and 7 d after the start of the CORT injection. In contrast, the CORT treatment significantly decreased the plasma levels of fT_3_ hormone at 7 d after the start of injection (2.0 pg/ml) when compared to 0 and 3 d after the start of injection (5.5 and 5.0 pg/ml, respectively; P<0.05). Seven days following the termination of the CORT injection (d 14), the fT_3_ levels returned to similar levels of their controls (5.5 and 5.8 pg/ml at 14 d after the start of injection for CORT and CONT groups, respectively; Fig 5-B). On the other hand, the plasma levels of AST in the CORT group were significantly higher at 3 and 7 d after the start of injection (131.0 and 137.4 U/ml, respectively) and also at 7 d after the interruption of CORT treatment (147.0 U/ml) than those levels at the start of the CORT injection (93.6 U/ml at 0 d of injection); however, this increase was significant only when compared to the CONT group at 7 d after the start of the CORT injection (Fig 5-C). As represented in Fig 5-D, the ALT in the CORT group was significantly higher after 3 and 7 d compared to 0 d of starting the injection course (21.2 and 25.0 vs. 14.2 U/ml, respectively). The ALT level decreased after the recovery period of chickens (13.4 U/ml at 14 d after the start of the CORT injection). The differences in ALT between CORT and CONT groups were significant only at 3 and 7 d after the start of the treatment (P<0.05).

### Quantitative real-time PCR analysis

**Fig 6:**
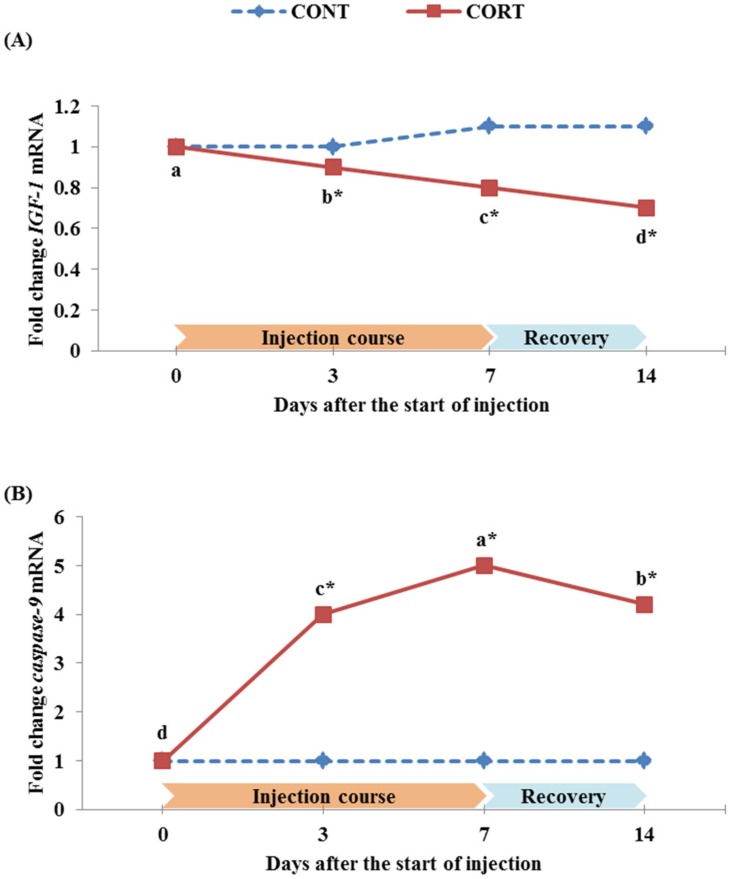
Effect of a 7 d course of daily saline (CONT) or corticosterone at dose of 5 mg/kg BW (CORT) injections on the relative expression of IGF-1 (A) and caspase-9 (B) genes in broiler chickens during the treatment course (0, 3 and 7 d after the start of injection) and one week after cessation of the treatment (14 d after the start of injection). Data express the mean ± SEM (n = 5). Means within a treatment group with different letters (a, b, c) are significantly different at P<0.05.*Significant difference between treatment groups within each time of the treatment course (P<0.05).

The relative expression of examined genes IGF-1 and caspase-9 in broiler chickens during the 7 d course of daily corticosterone injections and after 1-week recovery period post-treatment are shown in Fig 6. The current data showed that the relative expression of IGF-1 gene decreased significantly (P<0.05) in the treated broiler chickens by 0.11 and 0.22 fold at 3 and 7 d after the start of the CORT injection (Fig 6-A). This decrease continued to exist even after the termination of the CORT injection course (0.36 fold at the end of recovery period). In contrast, the expression of caspase-9 gene increased significantly (P<0.05) by 4.0 and 5.0 fold at 3 and 7 d after the start of the CORT injection (Fig 6-B). At the end of recovery period (d 14), the expression of caspase-9 gene decreased during the recovery period, but it still remained significantly (P<0.05) higher than the controls by 4.2 fold.

### Morphological detection of cell death categories

**Fig 7:**
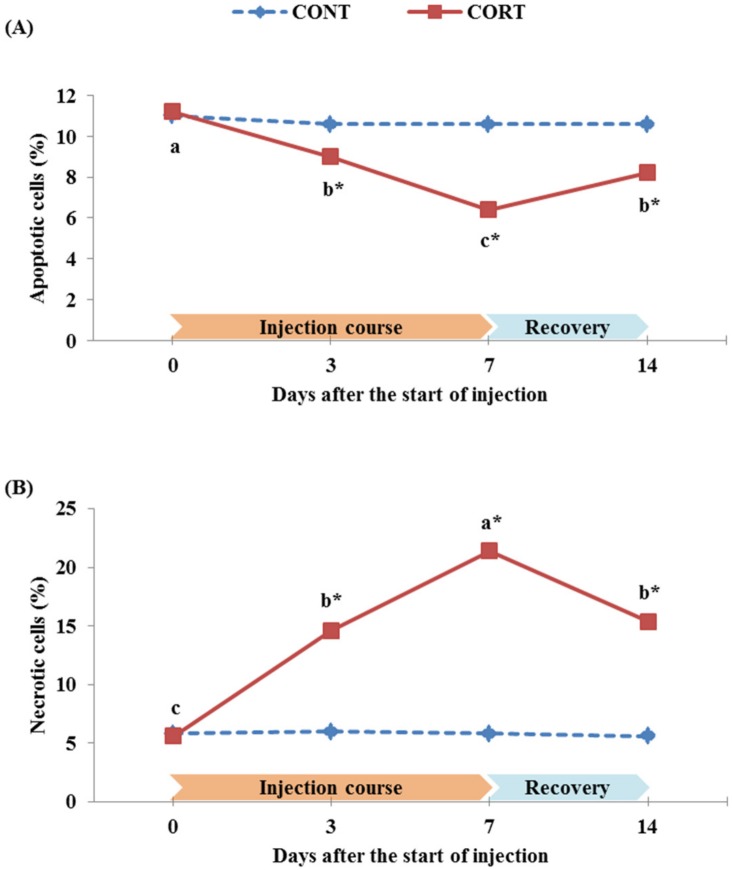
Effect of a 7 d course of daily saline (CONT) or corticosterone at dose of 5 mg/kg BW (CORT) injections on the % of apoptotic (A) and necrotic (B) cells in broiler chickens during the treatment course (0, 3 and 7 d after the start of injection) and one week after cessation of the treatment (14 d after the start of injection). Data express the mean ± SEM (n = 5). Means within a treatment group with different letters (a, b, c) are significantly different at P<0.05.*Significant difference between treatment groups within each time of the treatment course (P<0.05).

The results of splenic cell death category rates in broiler chickens during the 7 d course of daily corticosterone injections and one week after the post-treatment recovery period are illustrated in Fig 7. The % of total apoptotic and necrotic cells examined in CONT chicken group ranged between 10.6–11.0% and 5.6–6.0%, respectively. The chickens treated with corticosterone showed a significantly (P<0.05) lower % of apoptotic cells during the CORT injection course when compared to its control (9.0% and 6.4% vs. 11.2% at 3 and 7 d vs. 0 d after the start of the CORT injection, respectively; Fig 7-A). In contrast, a significantly (P<0.05) higher % of necrotic cells was observed after CORT injection when compared to its control (14.6% and 21.4% vs. 5.6% at 3 and 7 d vs. 0 d after the start of the CORT injection, respectively; Fig 7-B). At the end of recovery period (d 14), the apoptotic cells increased again (8.2%) while the necrotic cells decreased (15.4%) in the CORT group, but it remained significantly different from their controls (10.6% apoptosis and 5.6% necrosis in the CONT group; Fig 7).

## Discussion

Any type of stress triggers many biological mechanisms in the body of animals and humans to re-establish the homeostatic conditions and maintain the physiological activity [46]. The chicken is an important animal model well characterized in many biological aspects and bridges the evolutionary gap between mammals and other vertebrates [47]. At the same time, the massive meat production of broiler chickens make them continuously exposed to a wide range of potential stressors [1]. In our previous work [48], we found that endotoxin stress by E. coli infection to laying chickens markedly increased plasma corticosterone concentration by approximately 3 times more than normal chickens only 3 hr after E. coli injection. Such results and previous reports concluded that various forms of stress directly or indirectly activate the hypothalamic-pituitary-adrenal (HPA) axis and the release of corticosterone into blood [2].

Several investigations administrated the corticosterone in chicken diets at a concentration of 20–30 mg/kg [49,50] or in drinking water at 5–20 mg/L [51,52] to induce a stress status. Other investigations injected the corticosterone at doses of 4–6 mg/kg body weight once a day for 7 days to mimic acute stress in broiler chickens [3,53,54]. In this research, we directly injected broiler chickens with corticosterone at a dose of 5 mg/kg BW over 7 consequent days to simulate the stressful situation in these chickens; focusing on a wide range of expected responses at physiological, immunological and molecular levels throughout one-week of daily intramuscular CORT injection and after one-week of the cessation of CORT injection to test the ability of broilers to recover after termination of the stress condition.

In agreement with previous studies [3–7,29], all growth performance measured in this experiment was negatively affected by the CORT treatment (Fig 1). The final BW, DG, FI and FC of broiler chickens during the week of the CORT injection course significantly decreased when compared with the controls because elevated corticosterone levels inhibits anabolic processes and suppress appetite [55]. It was also reported that CORT may suppress growth performance by reducing the absorption of feed through the small intestine [56]. Furthermore, we found that growth performance traits, except FC, of broilers from CORT group remained lower than that of broilers from CONT group after 7 days of cessation of the CORT treatment (Fig 1). In this context, Yang *et al*. [3] found that feeding CORT-treated broiler chickens a high-energy diet did not compensate for the adverse effects of CORT-induced stress on growth rates. It means that CORT effect may be extended for a long time on stressed broilers even after the termination of CORT treatment or stress condition.

The relative weight of liver in broilers from CORT group was higher (P<0.05) than that in broilers from CONT group at 3 and 7 d after initiation of the CORT injection (Fig 2-A). Similar results were obtained by Jiang *et al*. [6] and Lin *et al*. [55]. The increase in liver weights after CORT injection is apparently due to the hepatic lipogenesis and concomitant accumulations of lipids in liver tissues [57–59].

On the contrary, the CORT treatment significantly decreased the relative weight of thymus, bursa and spleen (Fig 2-B, 2-C and 2-D, respectively). The decrease in relative weights of such immune-organs by CORT administration was also observed in other studies [3,9], and confirmed the sensitivity of immune-organs to corticosterone [60–62]. It was found that the administration of glucocorticoids resulted in a rapid degeneration of primary lymphoid tissues, such as thymus and bursa, due to their apoptotic effects [63,64]. However, the induced apoptosis may vary depending on the cell type and the expression levels of glucocorticoid receptors in these organs [65,66]. A study conducted by Schaumburg and Crone in 1971 [67] demonstrated that bursa lymphocytes in chickens contain higher levels of glucocorticoid receptors than those of the thymus, which makes the bursa more susceptible to the effects of glucocorticoids. Conversely, other studies reported that the thymus is one of the body tissues with the highest content of glucocorticoid receptors [68], and that the immature thymocytes show a higher glucocorticoid receptor density than matured thymocytes in human thymus [69]. In the present study, we noted that the decrease in thymus relative weight appeared early at 3 d after the start of the CORT treatment; moreover, the negative effect of CORT treatment on the thymus relative weight continued at 7 d after the start of the CORT treatment, and did not recover even 1 week after cessation of the 7-days CORT treatment (Fig 2-B). In contrast, the negative effect of CORT treatment on the relative weights of bursa and spleen appeared after a longer time of daily CORT injection (at 7 d after the start of CORT injections, not earlier), and these birds were able to recover these organs near to their normal weights as recorded in CONT broilers after cessation of the treatment (Fig 2-C and 2-D). Although we did not determine the expression of corticosterone receptors in thymus, bursa and spleen in this study, it is likely that these receptors might control the variance in the relative weight suppression observed among these organs after CORT treatment.

It has been emphasized that elevated plasma corticosterone and increased circulating H/L ratio are the two most accepted indicators of the stress condition in birds [11]. In a series of experiments conducted by Shini *et al*. [9,51,70,71] on young chickens, it was demonstrated that plasma corticosterone concentration and H/L ratio significantly increased post administration with exogenous corticosterone, and this was associated with a decreased peripheral lymphocyte count and increased peripheral heterophil count, whereas the total circulating leukocyte number decreased. In the present study, the TWBC’s was significantly (P<0.05) decreased by the CORT treatment (37.8 vs. 59.6 x10^3^/μl and 28.0 vs. 62.1 x10^3^/μl at 3 and 7 d after the start of the treatment for CORT vs. CONT group, respectively; Fig 3-A). The H/L ratio was significantly (P<0.05) increased at the same times (0.64 vs. 0.30 and 0.91 vs. 0.34 H/L ratio at 3 and 7 d after the start of the treatment for CORT vs. CONT group, respectively; Fig 3-B). In our experiment, the negative effect of CORT on the TWBC’s and H/L ratio in CORT-treated chickens, during the week of the injection course, was followed by a gradual improvement that remained low after 1 week of cessation of the CORT injections compared to its values in CONT chickens (the TWBC’s increased again to 35.2 x10^3^/μl vs. 60.8 x10^3^/μl and the H/L ratio decreased again to 0.65 vs. 0.35 in CORT group vs. CONT group, respectively; Fig 3). These results are in accordance with those obtained by other researchers [14,51,71] who studied the administration of CORT in diets and drinking water of chickens and obtained a high H/L ratio with a high plasma corticosterone values even after interruption of CORT supplementation. These observations assumed that corticosterone directly causes a redistribution of leukocyte components in the blood and a proportional change in the H/L ratio to multiply the cells required for the nonspecific immune response such as heterophils [72].

On the other hand, the CORT treatment negatively affected the lymphocyte proliferation, another important index to stress and correlated to cell mediated response in chickens [9,71]. These findings are confirmed with our results since the SI of T cells was decreased (P<0.05) by the CORT treatment from 5.01 at 0 d of the injection course to 2.25 and 1.74 at 3 and 7 d after the start of the injection course, respectively (Fig 4-A). The SI of B cells was also decreased (P<0.05) from 2.56 at 0 d of the injection course to 0.92 and 0.65 at 3 and 7 d after the start of the injection course, respectively (Fig 4-B). In addition, the SI of T and B lymphocytes were still lower (P<0.05) in the CORT group than in the CONT group after the recovery of chickens and termination of the CORT treatment (Fig 4). In birds, lymphocytes are specifically differentiated into T and B cells during development in the thymus and bursa, respectively [73]. Therefore, the low production of T and B cells in CORT-treated broilers in this study may be due to the decrease in the relative weight of these two organs (thymus, as shown in Fig 2-B; and bursa, as shown in Fig 2-C). It may also be attributed to the decrease in the number of lymphocytes in the CORT-treated chickens (inducing high H/L ratio, as shown in Fig 3-B). Previous studies on captive birds have shown that T-cell mediated acquired immunity is directly and positively linked to food intake and body mass [74], which have been negatively affected by the CORT treatment in our study (as shown in Fig 1) and, consequently, decreased the stimulation of T cells. Moreover, the chicken lymphocyte proliferation may be inhibited by corticosterone due to lipid peroxidation of cellular membranes of these cells [75].

In this study, some plasma biochemical constituents were affected by the corticosterone treatment. The TP significantly (P<0.05) increased from 2.98 g/dl before starting the CORT injection to 4.86 and 4.74 g/dl at 3 and 7 d after the start of the CORT injection compared to the CONT group (Fig 5-A). Similar results were obtained by a recent study [3], in which the CORT treatment enhanced the total plasma protein levels in broiler chickens. The significant increase in plasma protein of the CORT-treated broilers was accompanied by a significant decrease in the body weight (Fig 1) and the relative weights of immune-organs studied in our experiment (Fig 2). This observation is supported by findings of many authors [18,19,76], who reported that glucocorticoids or its synthetic compounds like dexamethasone retard the growth of skeletal muscles by suppressing protein synthesis and increasing protein catabolism in chickens [77]. The apparent elevation of plasma protein in the CORT-treated chickens may also be a result of the concomitant enhancement in protein-derived gluconeogenesis which is activated by glucocorticoids [78]. In addition, insulin-like growth factors and the thyrotrophic [triiodothyronine (T_3_) and thyroxine (T_4_)] axis are considered to be prerequisites for the normal growth and development [27]. In our study, we measured the plasma free T_3_ hormone levels and we found a significant decrease after initiation of the CORT treatment at 7 d (2.0 vs. 5.6 pg/ml for CORT vs. CONT group, respectively; P<0.05), and then it returned to similar levels of CONT group after cessation of the treatment (Fig 5-B). Previous studies indicated that exogenous CORT administration depressed circulating T_3_ levels by the inhibition of T_4_ synthesis and peripheral deiodination [27,28]. However, the decrease in fT_3_ levels in CORT-treated chickens was not observed before the 7^th^ d of the course of the treatment in the present study, indicating a slow response and adjustment of T_3_ levels after short-term administration of CORT [28]. In addition to the effects on plasma protein and free T_3_ hormone, CORT treatment significantly (P<0.05) increased the plasma levels of AST and ALT enzymes particularly during the week of the CORT injection course (Fig 5-C and 5-D, respectively). Although we observed a higher relative weight of liver in broilers from the CORT group compared to CONT group (Fig 2-A), the increase observed in plasma ALT and AST levels in CORT group demonstrated the failure in liver function caused by CORT treatment [20]. As previously mentioned, CORT increased the relative weight of liver mainly due to the increase of lipid deposition, and this in turn induces an impairment of liver tissues and a leakage of enzymes from cell membranes into the blood circulation [79].

There is a strong evidence that the administration of IGF-1 stimulates growth rate and protein synthesis [80] by its role in mediating growth hormone and thyroid hormones [81]. One mechanism by which glucocorticoid hormones depress growth in addition to reduced feed intake in chickens is by depressing circulating concentrations and hepatic expression of IGF-1 [22,29]. In line with these reports, the current data showed that the relative expression of hepatic IGF-1 gene significantly (P<0.05) decreased in the CORT-treated broiler chickens at 3 and 7 d after initiation of the CORT injection by 0.11 and 0.22 fold, respectively. Furthermore, we found that the relative expression of IGF-1 in CORT-treated broiler chickens continued to decrease significantly (P<0.05) after the termination of the CORT injection course (down-regulation by 0.36-fold at 14 d compared to 0 d of the CORT treatment; Fig 6-A). This inhibition of recovery for liver IGF-1 mRNA after termination of the CORT treatment was also observed in fasting-stressed chickens [82] and rats [83] after re-feeding. The data obtained from growth performance also confirmed the incapacity of the CORT-treated broilers to recover to the normal rates of broilers in the CONT group (Fig 1).

The other responsible mechanism for growth inhibition by glucocorticoid hormones in chickens is the proteolytic action of caspases on specific cell substrates [33,34]. This process has been studied in some details [84]; glucocorticoid signaling increases the expression of the pro-apoptotic Bcl-2 family member, which can activate the pro-apoptotic proteins Bax/Bak to disrupt mitochondrial membrane potential, resulting in the release of cytochrome c and other apoptogenic proteins. Consequently, caspase-9 and subsequent effector caspase-3 are activated, finally leading to apoptosis. In the present study, we focused on evaluating the relative expression of caspase-9 gene in the spleen of broiler chickens after corticosterone injection because it is an important member of caspases that is responsible for both initiating and executing apoptosis [35,36]. The expression of caspase-9 gene significantly (P<0.05) increased by 4.0 and 5.0 fold at 3 and 7 d of the course of the treatment, respectively (Fig 6-B). At the end of the recovery period, the expression of caspase-9 gene started to decrease but remained significantly (P<0.05) higher, in the treated chickens, than in the control chickens by 4.2 fold. Generally, Collier *et al*. [85] reported that endogenous glucocorticoids caused an increase in the splenic apoptotic cells in stressed-mice. Our results concluded that CORT inhibited the growth in treated chickens by both down-regulating hepatic IGF-1 gene expression and up-regulating splenic caspase-9 gene expression.

Although apoptosis can occur as a normal and beneficial defense mechanism in most organisms, necrosis can also be seen as a part of cellular death during stressful stimulation but it is considered as an unnatural cell death process [86]. The necrotic pathway form of cell death programs is accompanied by a rapid collapse of plasma membrane and it is independent of expression of new genes [38]. In the present study, we targeted at providing further information about the necrotic versus apoptotic pathways induced by CORT treatment in broilers as an expected result of exposure to stress. As a reliable criterion for distinguishing between apoptosis and necrosis, we morphologically detected the apoptotic and necrotic cells in the spleen of treated and untreated broiler chickens with CORT using fluorescent microscope after labeling with AO/EB dyes. We found that corticosterone significantly (P<0.05) decreased the % of apoptotic cells from the range of 10.6–11.2% in CONT group down to 9.0%, 6.4% and 8.2% in CORT group at 3, 7 and 14 d after the start of the injection course, respectively (Fig 7-A). On the contrary, the corticosterone treatment significantly (P<0.05) increased the rate of necrotic cells from the range of 5.6–6.0% in CONT group up to 14.6%, 21.4% and 15.4% in CORT group at 3, 7 and 14 d after the start of the injection course, respectively (Fig 7-B).

To the best of our knowledge, these results are the first reports concerning the apoptotic and necrotic pathways comparison after CORT treatment in broiler chickens. A study that most closely resembled our examination of apoptosis versus necrosis with CORT treatment was examining Leydig cells treated with CORT in rats [39]; in which signs of necrosis were seen more than apoptosis in treated cells with high doses of CORT compared to untreated cells or cells treated with lower doses of CORT. In another study conducted on rats [40], they reported cells of apoptosis and necrosis as 10.51% and 1.98% at 10^−6^ mol/L of CORT vs. 4.87% and 26.39% at 10^−3^ mol/L of CORT, respectively. Whereas the apoptotic pathway can potentially be modulated to maintain cell viability under stress condition [87], it seems that CORT treatment blocks this pathway. The high necrosis vs. low apoptosis incidence in splenic cells that we obtained during and after the daily 7-d CORT injection course in broiler chickens indicated that the severe stress and long-term CORT administration increases the possibility of tissue and cell damages, and induces cell death in an irreversible process or incompatible survival recovery [86,88]. This may also explain why the reduction in IGF-1 (Fig 6-A) and the increase in caspase-9 (Fig 6-B) expression in the treated chickens remained high and did not recover to its normal expression in the control chickens after cessation of the CORT treatment in our study.

In conclusion, the current study provides overall view of physiological challenges modulated in broiler chickens under mimicked stressful condition by CORT treatment. The growth performance was negatively affected by the CORT injection accompanied by low concentration of free T_3_ hormone in plasma and down-regulation of hepatic IGF-1 gene. Failure in liver functions was seen with CORT treatment by the increase in liver weight with an increase in plasma levels of total protein, and AST and ALT enzymes. Moreover, the relative weight of immune-organs decreased in the CORT-treated chickens with low counts of TWBC’s and lymphocyte proliferation, while the H/L ratio increased; indicating, as expected, immunosuppressive effect for CORT. Furthermore, high expression of caspase-9 gene was observed in the bursa of CORT-treated chickens, however, it was associated with high necrotic vs. low apoptotic cell death pathway in the spleen. After termination of the CORT treatment in broilers, most of these aspects remained negatively affected by CORT and did not recover to its normal status. Such information provides more understanding regarding the pathways of stress in broilers and may set the means through which a chicken is able to mount a successful defense against stress.

## Supporting information

S1 Table Effect of a 7 d course of daily saline (CONT) or corticosterone at dose of 5 mg/kg BW (CORT) injections on apoptotic categories of splenic cells in broiler chickens during the treatment course (0, 3 and 7 d after the start of injection) and one week after cessation of the treatment (14 d after the start of injection). (DOCX)

S1 Fig Visual examples for apoptotic categories determined morphologically under fluorescent microscope after labeling with acridine orange and ethidium bromide (AO/EB) dyes. Images represent samples of splenic cells from broiler chickens treated with a 7-d course of daily saline (CONT) or corticosterone at dose of 5 mg/kg BW (CORT) injections during the treatment course (0, 3 and 7 d after the start of injection) and one week after cessation of the treatment (14 d after the start of injection). Viable cells appear green, apoptotic cells appear yellow, and necrotic cells appear orange/red colored. Higher expression of necrosis can be seen in CORT 3, CORT 7 and CORT 14 images. Scale bars: 100 μm.
(TIF)
