# Supplementary material for: Comprehensive growth performance, immune function, plasma biochemistry, gene expressions and cell death morphology responses to a daily corticosterone injection course in broiler chickens
Source: PLoS One. 2017 Feb 24;12(2):e0172684. doi: 10.1371/journal.pone.0172684 (PMC5325522; doi:10.1371/journal.pone.0172684)
Supplement: S1 Table — (DOCX) [file pone.0172684.s001.docx]

**S1 Table. Effect of a 7 d course of daily saline (CONT) or corticosterone at dose of 5 mg/kg BW (CORT) injections on apoptotic categories of splenic cells in broiler chickens during the treatment course (0, 3 and 7 d after the start of injection) and one week after cessation of the treatment (14 d after the start of injection).**

| **Treatment** | **No. of animals** | **No. of cells Analyzed** | **Cell death categories (%)^1^** | | | **Total cell death (%)^1^** |
| --- | --- | --- | --- | --- | --- | --- |
|  |  |  | **Live cells** | **Apoptotic cells** | **Necrotic cells** |  |
| **CONT** |  |  |  |  |  |  |
| **0 d** | 5 | 100 | 83.2±0.49 | 11.0±0.32 | 5.8±0.20 | 16.8±0.49 |
| **3 d** | 5 | 100 | 83.4±0.75 | 10.6±0.60 | 6.0±0.32 | 16.6±0.75 |
| **7 d** | 5 | 100 | 83.6±0.75 | 10.6±0.51 | 5.8±0.37 | 16.4±0.75 |
| **14 d** | 5 | 100 | 83.8±0.74 | 10.6±0.51 | 5.6±0.25 | 16.2±0.74 |
| **CORT** |  |  |  |  |  |  |
| **0 d** | 5 | 100 | 83.2±0.58^a^ | 11.2±0.37^a^ | 5.6±0.25^c^ | 16.8±0.58^c^ |
| **3 d** | 5 | 100 | 76.4±0.51^b*^ | 9.0±0.32^b*^ | 14.6±0.25^b*^ | 23.6±0.51^b*^ |
| **7 d** | 5 | 100 | 72.2±0.37^c*^ | 6.4±0.25^c*^ | 21.4±0.40^a*^ | 27.8±0.37^a*^ |
| **14 d** | 5 | 100 | 76.4±0.40^b*^ | 8.2±0.37^b*^ | 15.4±0.25^b*^ | 23.6±0.40^b*^ |

**^1^**results were expressed as least square means ± SEM.

^a,b,c^ Means within a treatment group with unlike superscript letters are significantly different at P<0.05.

^*^Significant difference between treatment groups within each time of the treatment course (P<0.05).
