# Supplementary figures and images for: Comprehensive growth performance, immune function, plasma biochemistry, gene expressions and cell death morphology responses to a daily corticosterone injection course in broiler chickens
Source: PLoS One. 2017 Feb 24;12(2):e0172684. doi: 10.1371/journal.pone.0172684 (PMC5325522; doi:10.1371/journal.pone.0172684)

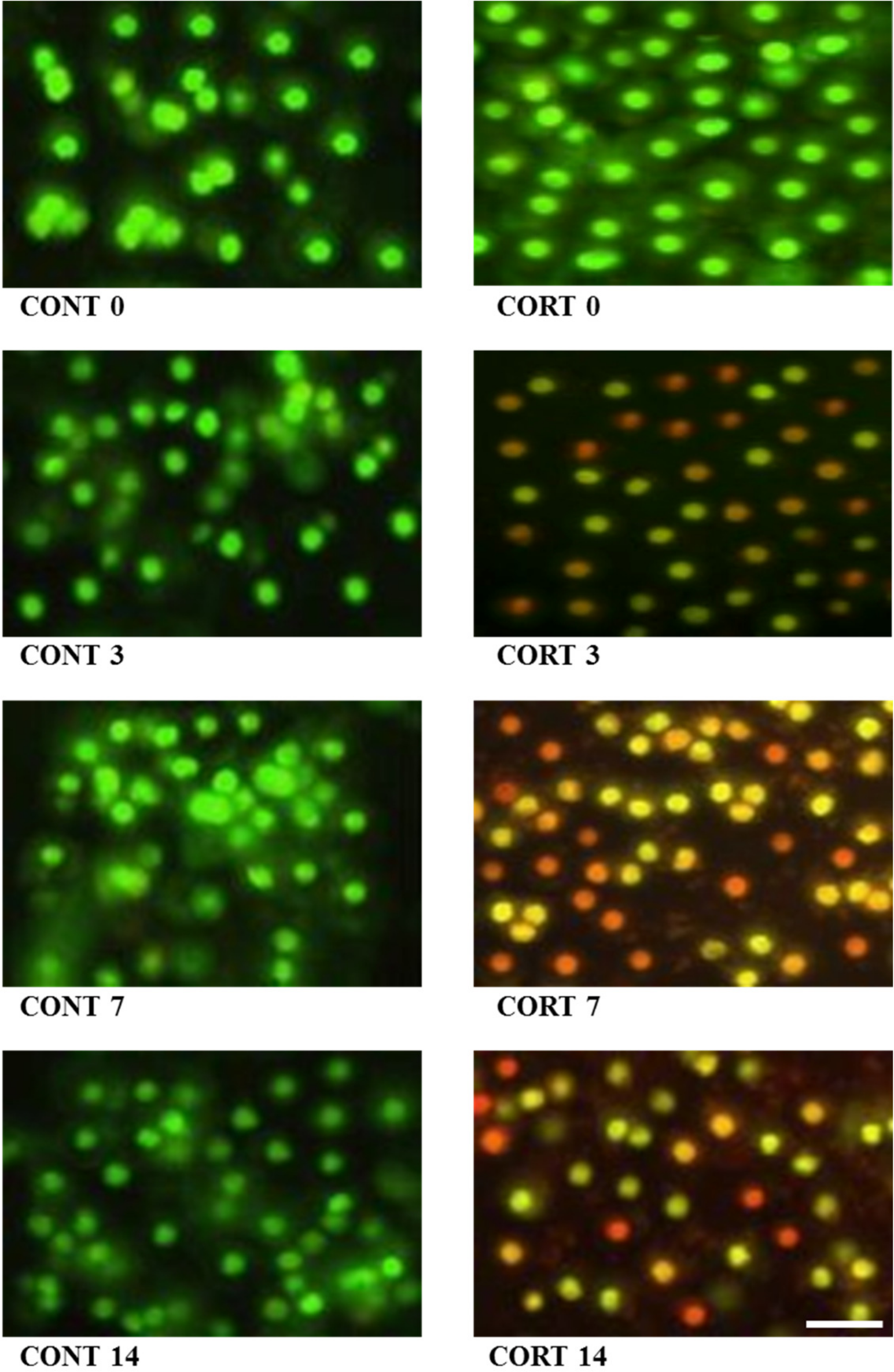

Supplement: S1 Fig — Images represent samples of splenic cells from broiler chickens treated with a 7-d course of daily saline (CONT) or corticosterone at dose of 5 mg/kg BW (CORT) injections during the treatment course (0, 3 and 7 d after the start of injection) and one week after cessation of the treatment (14 d after the start of injection). Viable cells appear green, apoptotic cells appear yellow, and necrotic cells appear orange/red colored. Higher expression of necrosis can be seen in CORT 3, CORT 7 and CORT 14 images. Scale bars: 100 μm. (TIF) [file pone.0172684.s002.tif]
